# Median Effective Dose of Dexmedetomidine Inducing Bradycardia in Elderly Patients Determined by Up-and-Down Sequential Allocation Method

**DOI:** 10.7150/ijms.71380

**Published:** 2022-06-13

**Authors:** Hua Yang, Yu Fu, Fang Deng, Yun Shao, Yu-Gang Lu, Jin-Chao Song

**Affiliations:** 1 Department of Anesthesiology, Shidong Hospital Affiliated to University of Shanghai for Science and Technology, Shanghai, China.; 2 Department of Anesthesiology, Shanghai Pulmonary Hospital, School of Medicine, Tongji University, Shanghai, China.

**Keywords:** dexmedetomidine, bradycardia, elderly patient, intravenous anaesthesia, adverse effects

## Abstract

**Purpose:** When dexmedetomidine is used in elderly patients, high incidence of bradycardia is reported. Given age-related physiological changes in this population, it is necessary to know the safety margin between the loading dose of dexmedetomidine and bradycardia. Therefore, we conducted this study to investigate the median effective dose (ED50) of dexmedetomidine causing bradycardia in elderly patients.

**Methods:** Thirty patients with ages over 65 years undergoing elective general surgery were enrolled. The Dixon and Massay sequential method were applied to determine the loading dose of dexmedetomidine, starting from 1.0 µg/kg. The dose for the follow-up subjects increased or decreased according to the geometric sequence with the common ratio 1.2, based on the 'negative' or 'positive' response of the previous subject. Positive mean that the subject developed bradycardia during the test. Hemodynamic data including heart rate and systolic blood pressure were recorded. The level of sedation was assessed with the Observer Assessment of Alertness and Sedation Scale (OAA/S).

**Results:** Bradycardia occurred in 13 patients (43.3%). The ED50 of dexmedetomidine causing bradycardia was 1.97 µg/kg (95% CI, 1.53-2.53 µg/kg). OAA/S scores at 10 min after the beginning of the dexmedetomidine infusion and 10 min after the termination of dexmedetomidine administration showed no significant differences between the positive and negative groups (*P* > 0.05).

**Conclusion:** The ED50 of dexmedetomidine causing bradycardia in our cohort was higher than clinical recommended dose. A higher loading dose appears acceptable for a faster onset of sedation under careful hemodynamic monitoring.

**Trial registration:** ChiCTR 15006368.

## Introduction

Dexmedetomidine is a potent and highly selective α2-adrenoceptor agonist displaying anxiolytic, sedative, sympatholytic, and analgesic effects with minimum cardiovascular and respiratory depression 1. Neurophysiologically, sedation using dexmedetomidine approximates stage 2 non-rapid eye movement sleep and reduces the incidence of delirium 2-4. Moreover, the administration of dexmedetomidine during operation is linked with a reduction in the incidence of postoperative shivering, nausea and vomiting 5. Aside from all the advantages, dexmedetomidine also has its shortcomings, the most crucial of which are bradycardia and even, in rare cases, cardiac arrest, especially for elderly patients 6-10. A higher incidence of bradycardia in patients aged older than 65 years was reported by the registration documents of the European Medicines Agency and the US Food and Drug Administration 11.

The cardiovascular effects of dexmedetomidine begin with transitory hypertension after the administration of a loading dose, owing to the activation of α2B receptors, with consequent hypotension and bradycardia because of the reduction of sympathetic tone 12, 13. Specially, an upper dose of dexmedetomidine has more profound influence on hemodynamic parameters in elderly patients, and though the pharmacokinetic profile of dexmedetomidine was not altered by age 14. Thus, age-related physiological changes must be considered when administering intravenous dexmedetomidine to elderly patients.

The hemodynamic effects of dexmedetomidine after short-term (2, 5 or 10 min) infusions have been described and summarized at doses ranged from 0.25 to 4 μg/kg 15. It is known that dexmedetomidine had a significant dose-response relationship with bradycardia. However, the median effective dose (ED50) of intravenous dexmedetomidine conducing significant bradycardia in elderly patients remains undetermined.

The aim of this study was to determine the ED50 values of intravenous dexmedetomidine causing bradycardia in elderly patients (aged >65 years) by Dixon's up-and-down sequential allocation method.

## Materials and Methods

### Study design and patients

This study was approved by the Institutional Research Ethics Committee of Shidong Hospital Affiliated to University of Shanghai for Science and Technology. The trial was registered at Chinese Clinical Trial Registry (ChiCTR 15006368). Patients aged 65 years and over scheduled for general surgery under general anesthesia were considered eligible. Patients were excluded if they: (1) American Society of Anesthesiologists (ASA) physical status score ≥3, (2) allergies to any of the drugs used for anesthesia, (3) body mass index (BMI) ≤20 or ≥30 kg/m^2^, (4) taking hypnotics, opioid analgesic or antianxiety agents, (5) known or suspected heart failure (ejection fraction <40%), severe respiratory disease, renal or metabolic diseases, (6) heart rate (HR) < 60 bpm, sick sinus syndrome, atrioventricular block, arrhythmia or atrial fibrillation, (7) electrolyte disturbances, (8) could not complete the informed consent procedure independently. A total of 30 elderly patients undergoing general surgery from July to August in 2020 were enrolled in the study and written informed consent was obtained from each patient.

### Preoperative preparations and anesthesia protocol

After overnight fasting, the patients without any pre-medication were brought to a quiet operating room where a 20-gauge intravenous cannula was placed in the peripheral vein for Ringer's lactate solution at 15-18 ml/kg/h. All patients were monitored with electrocardiogram (ECG), HR, pulse oximetry (SpO_2_), and non-invasive blood pressure (BP).

The dexmedetomidine (Dexmedetomidine Hydrochloride Injection, 2 ml: 200 μg, Jiangsu Hengrui Pharmaceutical Co., Ltd.), diluted in 50 ml of saline 0.9% and delivered at an assigned rate by a Graseby 3500 syringe pump (SIMS Graseby Ltd., Herts, UK). The first patient was tested at a loading dose of 1.0 μg/kg in 10 min without maintenance dose, which was the recommended loading dose according to the instructions for dexmedetomidine. The dose for the follow-up subjects was determined by the response of the previous patient, using the modified Dixon's up-and-down method 16. The response of the patients was categorized as either 'negative' or 'positive'. Negative mean the patient did not develop bradycardia throughout the trial. Positive mean that the patient developed bradycardia during the test. The dose of dexmedetomidine for the next patient would be reduced if the previous patient had a positive result. On the contrary, the dose of dexmedetomidine for the next patient would be increased accordingly. The dose for the next subject increased or decreased step by step according to the geometric sequence with the common ratio 1.2. If the bradycardia occurs during the infusion of dexmedetomidine, the procedure was discontinued, and in case of persistence of severe bradycardia and/or accompanied by hypotension, a bolus of intravenous atropine 0.5 mg plus 200 ml of intravenous Ringer's lactate solution was administered. Oxygen was administered at a flow of 5 L/min by a nasal catheter during the study.

During 10 minutes after the termination of loading dose of dexmedetomidine infusion, early signs of hypotension, hypertension, bradycardia, respiratory depression, airway obstruction, apnoea, dyspnoea and/or oxygen desaturation were monitored continuously. At 10 minutes after the termination of the loading dose of dexmedetomidine administration, anaesthesia was induced with propofol (0.7-1.0 mg/kg), cisatracurium (0.2 mg/kg) and sufentanil (0.4-0.5 ug/kg). Endotracheal intubation was performed and mechanical ventilation began. Anesthesia maintenance was provided by sevoflurane.

### Measurements

The baseline (T_0_) of BP and HR were defined as the average of three consecutive measurements at the time when patients arrived in the operating room with supine position. Haemodynamic data were also measured at 3 min (T_1_), 6 min (T_2_) and 10 min (T_3_) after the beginning of the dexmedetomidine infusion, 3min (T_4_), 6 min (T_5_) and 10 min (T_6_) after the termination of dexmedetomidine administration and immediately before anesthesia induction (T_7_). Bradycardia was defined as HR less than 50 beats/min. Hypotension was defined as decrease in systolic blood pressure by more than 30% of the baseline value. Hypertension is defined as an increase in systolic blood pressure more than 30% of the baseline value. Other types of arrhythmia and hypoxemia were also recorded. The level of sedation was assessed at T_3_ and T_6_ using the Observer Assessment of Alertness and Sedation Scale (OAA/S) score 17.

### Statistical analysis

Owing to the characteristic of the up-and-down sequential method, the sample size was determined by the results of simulation study suggesting that 20-40 patients will provide stable estimate of ED50. Thus, a total of 30 patients were determined in this study. ED50 was estimated from the up-and-down sequences, using the modified Dixon's up-and-down method. The dosage of ED50 was determined from the midpoints of all independent pairs of patients who involve a crossover from 'negative' response to 'positive' response.

All statistical analyses were performed using SPSS, version 20.0 (SPSS Inc., Chicago, IL, USA). Data are expressed as mean (standard deviation, SD) for continuous variables, frequency (percentage) for categorical variables, or median (interquartile range, IQR) for continuous variables with skewed distribution, as appropriate. Nominal data were analyzed using the Chi-square test, normally distributed continuous data were analyzed using Student's t-test and non-normally distributed continuous data were analyzed using nonparametric Mann Whitney U test. For within-group comparison, the repeated-measures ANOVA with adjustment for multiple comparisons to control the type I error with Bonferroni test was used for normal data. Statistical significance was defined as P < 0.05.

## Results

**Figure 1:**
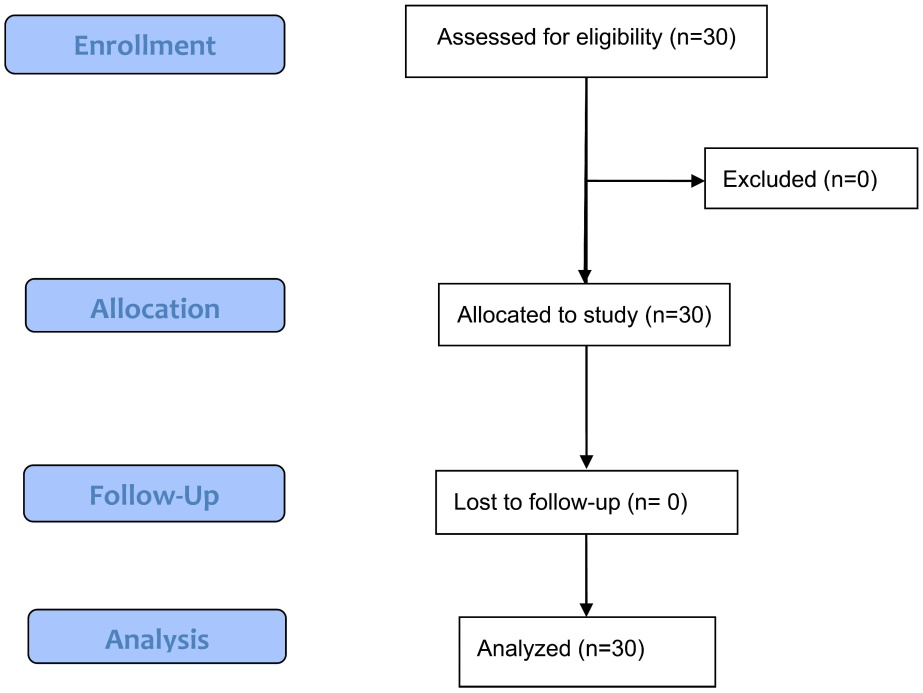
Flowchart of patients enrolled in the study.

The study flow diagram is presented in **Fig. 1**. The study was stopped after the enrolment of 30 consecutive patients. Patients' characteristics are presented in **Table 1**.

The defined positive outcomes took place in 13 patients, 11 of which had positive results during the dexmedetomidine infusion, and 2 of which had positive results after the infusion within 10 minutes. There was no significant difference in demographic data between the positive and negative groups (*P* > 0.05).

**Figure 2:**
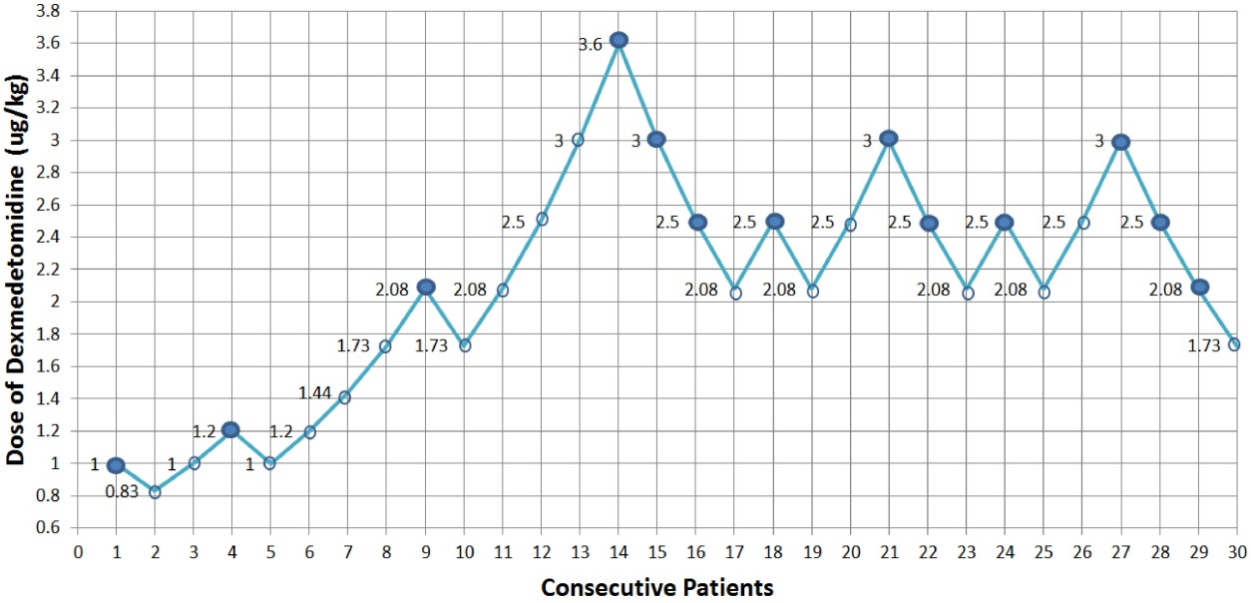
** Consecutive dose of dexmedetomidine for ED50 determination.** A positive dose is denoted by a solid circle; a negative dose is denoted by an open circle. Dexmedetomidine ED50 causing significant bradycardia was 1.97 µg/kg (95% confidence interval 1.53-2.53 µg/kg) calculated by Dixon and Massay formula.

**Figure 3:**
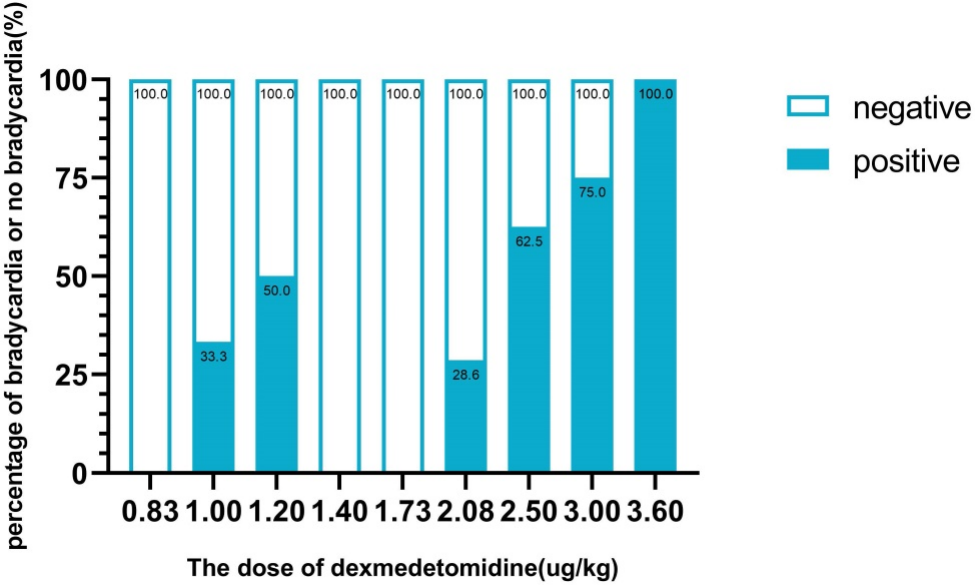
The percentage of bradycardia or no bradycardia at different doses of dexmedetomidine.

The sequence of the dose of dexmedetomidine is illustrated in **Fig. 2**. There were 8 pairs of reversal of sequence in this study, which showed that our sample size was enough. The assigned dexmedetomidine were 0.83 μg/kg, 1.0 μg/kg, 1.2 μg/kg, 1.44 μg/kg, 1.73 μg/kg, 2.08 μg/kg, 2.5 μg/kg, 3.0 μg/kg and 3.6 μg/kg. The ED50 of dexmedetomidine inducing bradycardia, by Dixon and Massay up-down sequential method, was 1.97 μg/kg (95% CI, 1.53-2.53 μg/kg) (**Fig. 2**). The percentages of different doses of dexmedetomidine inducing bradycardia are shown in **Fig. 3**.

OAA/S scores at T_3_ and T_6_ showed no significant differences between the 2 groups (*P* > 0.05). After dexmedetomidine administration, OAA/S scores of both groups at T_6_ were lower than those at T_3_ (*P* < 0.05) (**Table 2**).

**Figure 4:**
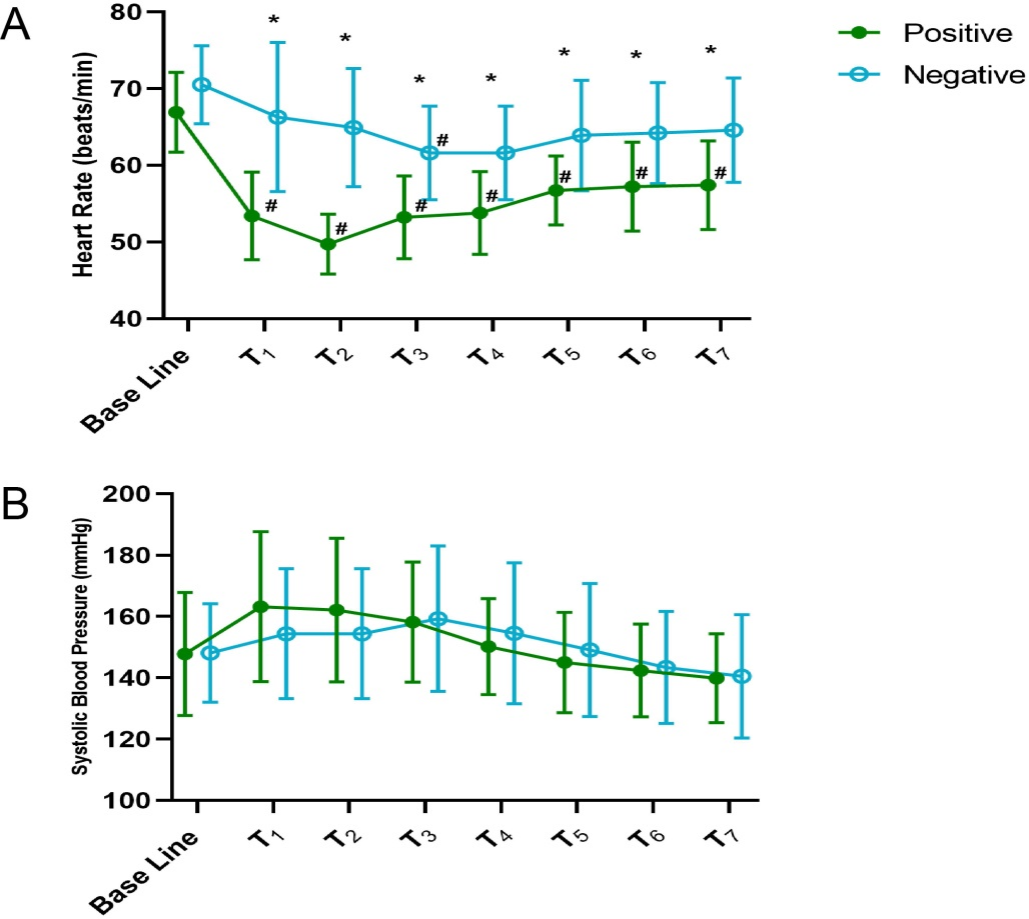
** The heart rate and systolic blood pressure in positive and negative groups. (A)** The heart rate at different times in the two groups. **(B)** The systolic blood pressure at different times in the two groups. Baseline: Before infusing the dexmedetomidine. *P < 0.05 compared between groups in the same period. ^#^*P* < 0.05 vs baseline within group.

The HRs were similar in all two groups at baseline (**Fig. 4A**). However, negative group showed significant increase in HR than positive group at T_1-7_ (*P* < 0.05). In addition, Comparing HR to baseline within positive group, HR was significantly lower at T_1-7_ (*P* < 0.05). Comparing HR to baseline within negative group, HR was only significantly lower at T_3_ (*P* < 0.05). Data regarding SBP are shown in **Fig. 4B**. SBP showed no significant difference in each comparison at the same period between the groups (*P* > 0.05). Comparing SBP to baseline within both groups, there were also no significant difference at each time point (*P* > 0.05).

The study was completed without any significant clinical complication. No episode of hypotension (systolic blood pressure decrease of 30% the baseline value), hypoxemia, or respiratory depression occurred during the study. The systolic blood pressure increased over 200 mmHg in 2 patients, but they were under 30% the baseline value. No patients needed atropine or ephedrine.

## Discussion

Bradycardia is a common adverse reaction of dexmedetomidine, which incidence varying from 10% to 30% depending on the dose of dexmedetomidine 15, 18. Age-adjusted dosing is not recommended, although caution is warranted as hemodynamic effect might be more pronounced in elderly patients 14. Previous studies 19 suggested that dexmedetomidine plays a valuable role in the multimodal administration of nonopioid agents which can reach the intraoperative hemodynamic stability and the antinociception traditionally obtained by opioids. However, the dose of dexmedetomidine is used for this purpose may promote risk of bradycardia during surgery 20.

It is likely that a lower dose might have prevented such an event, but which may not achieve the level of sedation required. For sedation in the operating room, a faster onset of effect from dexmedetomidine is often desired such as awake fibreoptic intubation 21, awake craniotomies 22, or rescue sedation under certain scenario 17. A loading dose of dexmedetomidine has been routinely administrated to gain a faster onset of effect and mitigate side effects by which infused over 10 minutes. To the best of our knowledge, the ED50 of intravenous dexmedetomidine conducing significant bradycardia in elderly patients (aged >65 years) remains undetermined.

The first question in this study sought to determine the ED50 values of intravenous dexmedetomidine causing bradycardia in elderly patients (aged >65 years). Studies have shown that loading doses of 0.5-1.0 µg/kg dexmedetomidine in 10 min, followed by an infusion at a rate of 0.2-0.7 µg/kg/h, provide effective sedation and be well-tolerated for elderly patients 23, 24. In this randomized up-down sequential allocation study, we found that the ED50 of intravenous dexmedetomidine causing bradycardia in elderly patients was 1.97 μg/kg (95% CI, 1.53-2.53 μg/kg). It has to be said that this is one unanticipated finding, which well above a loading dose of 1 μg/kg in 10 min that is recommended 14. What's more, when taking into account the possible effects of age-related physiological changes in elderly patients, a loading dose of like this in geriatric patients is not suggested. If a faster onset of sedation is needed, a slightly higher loading dose appears to be accepted for these patients under close supervision.

In the current study, the observation and recording of bradycardia is set before anesthesia induction, which advantage is the absence of other pharmacologic confounding factors, such as opioids and sevoflurane may have played the synergistic roles with dexmedetomidine. Previous studies 25 showed that the peak of HR decrease appeared at 3 minutes and carried on 11 minutes after infusing 0.25 μg/kg to 2.0 μg/kg of dexmedetomidine over 2 minutes in healthy adult volunteers. These changes were similar to our findings. In this study, 13 patients experienced bradycardia, 11 of which had positive results during the dexmedetomidine infusion, which was treated with suspending the infusion. Fortunately, no patients needed atropine or ephedrine throughout this study. In addition, the negative chronotropic properties of dexmedetomidine are well enough acknowledged that a clinical trial even describes the controlled hypotension use of dexmedetomidine during rhinoplasty 26. However, it is a nerve-wracking job to reduce the incidence of bradycardia while the hypotensive effect is preserved. The result of our study, therefore, has certain value in promoting the clinical application of dexmedetomidine.

Dexmedetomidine exerts sedative and anti-anxiety effects since acting on central pre- and postsynaptic a2-receptors in the locus coeruleus. After sedation, dexmedetomidine may also induce physiological sleep without obvious respiratory suppression 27. In this study, we observed similar levels of sedation between the two groups at 10 min after the termination of dexmedetomidine administration, which similar to the findings of Gu et al. 28 on the time to sedation.

Dexmedetomidine produces a typical biphasic effect on blood pressure, a transient increase followed by a longer lasting decrease in blood pressure 14. We also observed this biphasic response in SBP before anesthesia induction. This was thought to activation of ɑ2-receptor in the vascular smooth muscles, leading peripheral vasoconstriction and consequently hypertension. After a few minutes, the vasodilatation, presynaptic a2-adrenoreceptors inhibiting sympathetic release of catecholamines and the increased vagal activity, causing a hypotensive phase 29. Although the systolic blood pressure increased over 200 mmHg in 2 patients, the maximum changes in hemodynamics were all less than 30% of the baseline, which is generally considered acceptable.

Generally speaking, this study was an exploratory trial under the safety of all patients. All subjects had normal liver and kidney function without co-morbidities before enrollment. During dexmedetomidine infusion, all patients had continuous cardiac and respiration monitoring. In addition, the trial was conducted under the close supervision of experienced doctors. Finally, all subjects did not suffer further sequelae related to the dexmedetomidine in the recovery and postoperative periods.

There were several limitations in the current study. First, we did not record the hemodynamics data after anesthesia induction, which could be theoretically influenced by dexmedetomidine. Second, sedation in this study was only measured subjectively using OAA/S, lacking the bispectral index (BIS) monitoring during the infusion of dexmedetomidine. Third, the up-and-down method was used to determine the ED50 in the current study, which premise is that the dose-effect relationship is the traditional S-shaped curve, and often depended on a small sample size. But this did not provide reliable insight into the upper tail of the distribution, which may be imprecise 16. Besides, this study was launched in relatively healthy patients (normal renal and hepatic function) as well as these patients were not receiving any other common vasoactive agents (such as beta-blockers, calcium channel blockers, etc.) that might potentiate the hemodynamic effect of dexmedetomidine. This alleviates the generalization of these results to common practice. Additional studies, that different dexmedetomidine loading doses in different disease settings, are needed.

In conclusion, the ED50 of intravenous dexmedetomidine causing bradycardia in elderly patients was 1.97 μg/kg. A slightly higher loading dose appears to be accepted for a faster onset of sedation under careful hemodynamic monitoring. Moreover, the use of dexmedetomidine during daily practice should be cautiously evaluated for each patient.

## Figures and Tables

**Table 1:** Baseline characteristics of the patients

	Positive (n=13)	Negative (n=17)	*P*-value
Age, yr	73.5±6.4	74.1±8.2	0.836
Gender, male/female	8/5	10/7	0.880
Height, cm	169.0±7.6	163.5±8.5	0.077
BMI, kg/m^2^	23.1±2.7	23.4±3.9	0.776
ASA, Ⅰ/Ⅱ	8/5	11/6	0.858
Albumin, g/L	39.8±4.8	38.2±4.3	0.325
Bilirubin, μmol/L	16.0±6.2	14.3±5.4	0.441
ALT, U/L	28.2±13.7	21.8±20.7	0.342
AST, U/L	28.5±11.6	27.4±37.8	0.914
SCR, mmol/l	69.4±12.5	78.7±38.9	0.367
BUN, mmol/l	5.0±1.6	5.4±2.0	0.534
Baseline SBP, mmHg	147.8±20.1	148.1±16.1	0.968
Baseline HR, beats/min	66.9±5.2	70.5±5.1	0.075

Data are shown as mean ± SD. BMI, body mass index; ASA, American Society of Anesthesiologists; ALT, alanine aminotransferase; AST, aspartate aminotransferase; SCR, serum creatinine, BUN, blood urea nitrogen; SBP, systolic blood pressure; HR, heart rate. *P* value in Student's t-test, or χ^2^ test.

**Table 2:** Comparison of OAA/S score at T_3_ and T_6_ time points

	Positive (n=13)	Negative (n=17)	*P*-value
OAA/S score			
T_3_	5 (3.5, 5)	4 (2.5, 5)	0.503
T_6_	2 (2, 3)^*^	3 (2, 4)^*^	0.122

Data are presented as median (25th percentile, 75th percentile). OAA/S, Observer's Assessment of Alertness and Sedation Scale. ^*^P < 0.05 compared with the same group at T_3_.

## Abbreviations

ED50: median effective dose
ASA: American Society of Anesthesiologists
BMI: body mass index
HR: heart rate
ECG: electrocardiogram
BP: blood pressure
OAA/S: Observer Assessment of Alertness and Sedation Scale
BIS: bispectral index
